# A markerless, real-time, augmented reality-based surgical navigation system for neurosurgical biopsies

**DOI:** 10.1007/s00701-025-06750-x

**Published:** 2026-01-06

**Authors:** Annabel Groenenberg, Jan M. M. Heyligers, Bachtiar Burhani, Geert-Jan M. Rutten, Max M. Louwerse

**Affiliations:** 1 Department of General Surgery, Elisabeth-TweeSteden Hospital, 5022 GC Tilburg, The Netherlands; 2 Department of Neurosurgery, Elisabeth-TweeSteden Hospital, Tilburg, The Netherlands; 3 Department of Cognitive Science and Artificial Intelligence, Tilburg University, Tilburg, The Netherlands

**Keywords:** Augmented reality, Neuronavigation, Computer-assisted surgeries, Surgical navigation, Mixed reality, Frameless biopsies

## Abstract

**Purpose:**

Neurosurgical biopsies require high accuracy and precision and are executed with image-guided surgical navigation. The current state-of-the-art techniques require markers, are displayed on a 2D screen, and have a time-consuming setup. We propose an AR-driven surgical navigation method that automatically projects a 3D virtual overlay onto a patient in real-time, without the use of any markers.

**Method:**

Baseline accuracy of the proposed system and the StealthStation S8 was measured on a 3D printed human head phantom in a lab-based setting. For the measurements in the operating room, seventeen participants who underwent a neurosurgical biopsy with the StealthStation S8 were included. Prior to the clinical procedure, our proposed markerless AR system provided an automated three-dimensional virtual overlay onto the patient to the surgeon. By measuring the difference in the planned biopsy trajectory between the state-of-the-art StealthStation S8 and our experimental system, a comparison was made between the two systems.

**Results:**

The average clinical error for the entry point of the proposed system was 4.5 ± 2.2 mm, which is lower than the total error of the current clinical gold standard found in literature.

**Conclusion:**

The total error of the system proposed in this study reaches the gold standard for image-guided neuronavigation, in both lab-controlled and clinical settings. These initial results highlight the potential and advantages of AR over other methods, offering promising AR opportunities for future clinical applications.

## Introduction

Neurosurgical biopsies are the gold standard for determining the type of a found brain lesion. A biopsy needle is inserted into the brain to take a biopsy from the region of interest via a predetermined trajectory. To minimize complications, the lesion must be reached without causing damage to any critical structures. Therefore, it is essential that the actual biopsy trajectory matches the preoperatively planned trajectory. Image-guided surgical navigation is commonly used to execute this procedure, requiring both high accuracy and high precision for registration of the patient to the preoperative scan. This to ensure correct targeting of the structure with stable repeatability. However, these systems bring several technical and practical challenges.

First of all, the efforts required to calibrate the system are quite extensive. For instance, an anchor needs to be attached to the patient’s Mayfield clamp. This anchor needs to be visible at all times for the infrared camera that accompanies the navigational system, and it furthermore needs to be matched to an external instrument, e.g., a passive navigation probe. Moreover, for actual registration of the preoperative MR scan to the intraoperative situation, this specific tool needs to be traced along the patient’s face until the desired registration accuracy is achieved. Finally, before the patient can be covered with sterile cloths, this anchor first needs to be detached only to be reattached again afterwards. This intraoperative process is unnecessarily time consuming and cumbersome. On average the procedure consisting of setup, registration and instrument positioning of (robotic) navigation techniques takes approximately 15 minutes [18]. What is more, the registration accuracy often relies on external markers and on the static relationship between those markers and the patient. This introduces additional room for error. These uncertainties can cause the actual total error of the system when measured in a clinical setting (i.e., the total clinical error) to be larger than the target registration error created by the commercial navigation system itself, as shown in Table 1, showing the total clinical errors found when using the Medtronic StealthStation. Another disadvantage of the status quo is the constant switch between information display and work field for a surgeon. Once a proper registration has been achieved, operators must constantly refer to an external two-dimensional screen outside of their operating field to match the three-dimensional patient situation with the surgical planning.

**Table 1:** A summary of research that investigated the total clinical error between a target placement and final placement during neurosurgical procedures that use commercially available frameless stereotactic navigation systems

Research	System	Subject size *n*	Statistical parameter used	Total Clinical Error in mm
Furuse et al. 2023 [7]	Medtronic StealthStation S7	13	Median	3.3 (Front of face) 8.3 (Temporal left) 8.7 (Temporal right) 9.6 (Back of head)
Mongen et al. 2019 [19]	Medtronic StealthStation S7, S8 and BrainLab Curve	11	Mean	5.35 ± 1.64
Sao-Mai Sy Tay et al. 2022 [22]	Medtronic StealthStation S8 with Stealth Autoguide robotic arm	9	Mean	4.67 ± 0.27
Girgis et al. 2020 [8]	Medtronic StealthStation S7	13	Mean	7.8 ± 2.2

Augmented reality (AR) would solve many of the abovementioned problems and offers promising opportunities, as it may reduce errors and reduce procedural time, resulting in a more efficient use of hospital staff and equipment. The use of AR for clinical research has been around for decades, but it has really shown its promise since the introduction of head-mounted displays (HMDs) with optical waveguides such as HoloLens in 2016 [11, 15, 17]. Prior studies have shown the use of augmented reality for intraoperative guidance of neurosurgical procedures is a promising area of research, especially when combined with automated registration [2, 6, 11, 21]. However, most AR research uses marker-based technologies such as infrared (IR) reflective spheres or QR codes to achieve an intraoperative overlay [3, 21, 23, 24]. These methods bring several disadvantages as they require the introduction of additional material for registration. Although these methods may offer advantages over traditional techniques, they do not solve the time-consuming calibration and setup. Moreover, these markers must always be present and visible within the surgical field-of-view. Lastly, the static relation between the patient and markers must be ensured. This limits the applicability of marker-based methods [11, 16].

Here we describe and test a novel AR-based solution for neurosurgical biopsies called ARCUS (Augmented Reality for Clinical Understanding and Surgery). The ARCUS system is an AR-based 3D markerless navigation system that virtually overlays 3D models derived from a preoperative MR or CT scan directly onto the patient [9]. The registration of the preoperative scan to the live scene is fully automatic, with no need for any markers such as QR codes or reflective spheres. Instead, the system uses a depth sensor integrated into the hardware that matches the 3D models from the preoperative scan automatically to the 3D intraoperative scene. This innovative solution enables a surgeon to locate the entry point for the neurosurgical biopsy in 3D, directly onto the patient, within 6–10 seconds intraoperatively. Intraoperative setup simply requires donning the HMD, taking less than a minute. In this research, we define intraoperative setup time as the time required for a system to work after a patient has been brought under anesthesia. As a comparison, the intraoperative setup and registration time of the Medtronic StealthStation takes 15 to 20 minutes [18].

The markerless feature of our suggested AR solution does not suffer from the abovementioned problems and may therefore offer a promising method for intraoperative 3D navigation. This potential benefit was demonstrated in our prior research as well as in other studies that conducted similar research. However, in all these studies, the reported accuracy was still too large for clinical implementation, often reporting offsets nearing a centimeter [9, 11, 13, 14]. Recent improvements in our software for hardware interaction indicate improved accuracy and precision, as reported in our recent human specimen study [10]. In this research, we show an ex-vivo setup on 13 human specimen heads. On each head, 5 trajectories were planned and overlayed via an AR-based 3D hologram provided by our ARCUS system. A surgeon then placed 5 k-wires along each trajectory. Though initial limitations impacted the accuracy and precision of the current system, the experiments showed a median total clinical error of 3.91 ± 3.69 mm on par with, or even exceeding, the gold standard.

We still predict an impact of skin shift on our proposed solution, as described in Furuse et al. [7]. This phenomenon describes the distortion of the facial skin due to patient positioning during a procedure which introduces error in registration accuracy. The use of the facial landscape as reference landmark is therefore expected to result in a similar if not significantly lower total clinical error as found with the clinical gold standard [7]. This is because a larger number of landmarks are used for registration (e.g., the upper face) and because the proposed markerless approach needs no external reference markers, thereby limiting the introduction of additional errors.

In the current study, we first conducted a phantom test to determine the accuracy and precision of the clinical gold standard, the Medtronic StealthStation S8. Next, we performed a patient study assessing the accuracy and precision of ARCUS in an observational setting within the operating room (OR), on patients who needed to undergo a neurosurgical biopsy. The Medtronic StealthStation system was used as the measurement tool for this observational patient study.

## Method

To assess the accuracy and precision of the ARCUS system in an applied clinical setting, an observational study was conducted in the OR. A biopsy trajectory position overlayed by the ARCUS system was compared to the position defined by the Medtronic StealthStation that is used in our institution in daily practice and is a clinical gold standard for frameless navigation.

The current study involved two experiments to determine the offset of the proposed ARCUS system. First, we tested the reliability of the registration accuracy generated by the Medtronic StealthStation S8 in a lab-based setting. Because this system was used for measurements in the second experiment, the actual patient study, it was important to assess the influence of the system’s accuracy and precision on our in-patient measurement results.

The second experiment included an observational patient study. During this experiment, we exported the exact biopsy location planned on the MR scan from the clinical gold standard system, the Medtronic StealthStation S8, and used it as input in the ARCUS system. The correct biopsy location as given by ARCUS was next compared to the biopsy location as given by the clinical gold standard.

### Method and materials - StealthStation phantom test

**Fig. 1:**
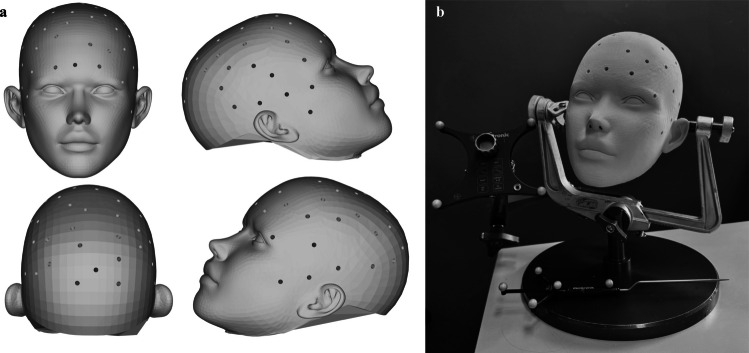
**a** On the left, shows a digital overview of the fifty-one conical points placed on the face-model. **b** On the right, shows the experimental setup for the 3D-printed face model as used for the phantom test

To assess the accuracy and precision of the used frameless stereotactic navigation of the Medtronic StealthStation S8, a phantom study was conducted. This was done by planning a biopsy trajectory along a direct physical trajectory that fits the Medtronic passive probe pointer. Once the pointer was positioned exactly in the physical trajectory, the resulting “Off-plan distance” of the system showed the system’s error. Fifty-one measurement points were incorporated into an open-source digital 3D model of a head, as shown in Fig. 1a [4]. These 51 conical holes served as true location to fit the Medtronic passive blunt probe into. This exact digital 3D model with all the conical holes was 3D printed, leaving us with a physical 3D model and its exact digital twin as output. Each point was positioned at the tip of an inverted cone 20 mm below the head’s skin surface, with the conical shape being the inverted shape of the measurement pointer, plus 0.2 mm margin. The setup is shown in Fig. 1b.

To be able to load the model into the StealthStation, the printed 3D model was next scanned in a CT scanner with a voxel size of 1.0*0.5*0.5 mm and loaded into the Medtronic StealthStation S8. Each trajectory was planned from the middle of the surface circle to the inverted tip of the cone, ensuring a true match.

**Fig. 2:**
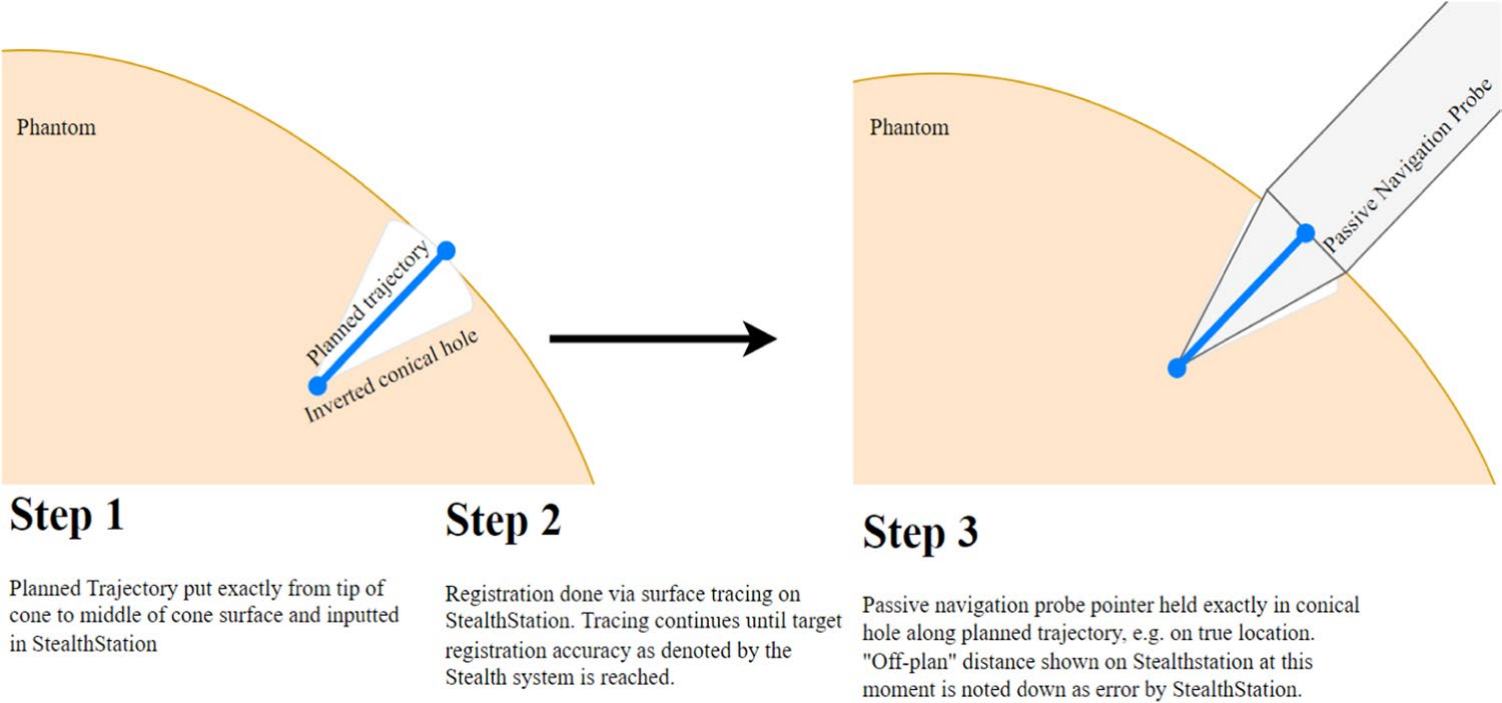
An overview of the protocol followed for the phantom study. Instead of one trajectory, fifty-one trajectories were inputted into the StealthStation system and measured for each registration accuracy

Subsequently, the planning was registered to the 3D-printed head on the StealthStation with varying within-system registration accuracies, as provided by the StealthStation. The measurements were repeated 5 times for different registration accuracies given by the system, varying from 0.5 mm to 2.5 mm with a step size of 0.5 mm. To achieve each exact desired registration accuracy by the system, the researcher traced the phantom surface until the targeted accuracy was reached. If the registration passed the desired output, e.g. becoming “too accurate”, the process was started over. Once a registration was achieved, the pointer was placed in the exact cone of each point and the off-plan distance was measured three times. The off-plan distance describes the shortest Euclidean distance of the instrument or probe tip used for measuring to the line of the biopsy trajectory that runs through the planned entry and target point. This was repeated for all fifty-one points on the head for each of the registration accuracies. An overview of the procedural steps is shown in Fig. 2.

### Participants - patient study

Seventeen adult patients were included in this study, exceeding the number of participants in similar studies (Table 1). These patients were selected for an elective biopsy of a brain lesion where the frameless, stereotactic brain biopsy technology with the passive navigation probe and IR-reflective marker spheres were used. The group consisted of six females and eleven males with an average age of 64 ± 10 years. Inclusion criteria consisted of a patient being at least 18 years of age, being capable of understanding the patient information and giving informed consent. The study was conducted in the Elisabeth-Tweesteden Hospital in Tilburg, the Netherlands.

### Materials - patient study

#### Software

Image segmentation was performed using 3D Slicer version 5.0.3 [1, 5]. Post-processing steps were conducted in Autodesk MeshMixer (Autodesk, Inc., San Francisco, USA) to allow for easy control of the triangle number per rendered 3D model. For the development of the ARCUS HMD application, the Unity Engine version 2020.3.46f1 (Unity Technologies, San Francisco, USA) was utilized, and several registration algorithms were included for both global and local registration of the target to the source model. The full process of the ARCUS algorithm was described in [9]. In summary, our system used the depth sensor of the HMD device to track the live 3D landscape intraoperatively. When a registration was required, it output live depth image data, which was then converted into a full 3D point cloud of the live depth landscape. This full landscape was then used for matching, also called registration. By first implementing TEASER + +, a robust, global registration algorithm that is proficient in dealing with noisy input data, followed by a local iterative closest point registration algorithm, a fast and fully automated matching was achieved [9, 20, 25, 26]. Data processing was conducted with R version 4.4.2 in RStudio version 2024.09.1 (RStudio, Boston, USA).

#### Hardware

Measurements on the OR were conducted using the Medtronic StealthStation S8 system (Medtronic, Dublin, Ireland) equipped with the included planar passive blunt probe. The StealthStation uses a large hardware station with a 2D screen and an infrared camera to detect and track infrared reflective spheres mounted onto an anchor and on the probe. These detected spheres are then used as references to match the preoperative MR scan of the patient to the actual patient. By tracing the probe along the skin of the patient, the surface is tracked and registered to the skin surface derived from the MR scan.

The ARCUS HMD application was deployed on a Microsoft HoloLens 2 headset (Microsoft, Redmond, USA), connected to a Razer Blade 17 laptop (Razer Inc, Irvine, USA) with a 12th Gen Intel(R) Core(TM) i9-12900H (2.50 GHz) processor and an Nvidia GeForce 3080 Ti GPU.

### Method - patient study

#### Preparation

**Fig. 3:**
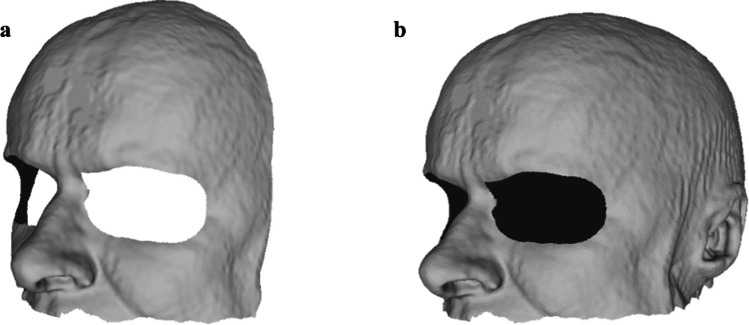
The two types of reference models used in this research. **a** Shows only the frontal face of the subject, while (**b**) also includes the rest of the skin on the head

As part of the standard treatment, each participant had to undergo a T1-weighted MRI-scan with an iso-spaced voxel size of 0.5*0.5*0.5 mm. This scan data was then pseudonymized and exported into Slicer 3D, version 5.0.3, where the skin surface and the lesion, if possible, were manually segmented with threshold-based segmentation. When the boundaries of a lesion were not properly visible, the lesion model was not segmented and displayed. These segmentations were subsequently post-processed for noise-reduction, including median smoothing with a window size of 3 mm, made solid with the “wrap solidify” extension and exported as 3D models. The participant’s skin surface 3D model that was used for visualization in a virtual overlay was reduced to 100,000 triangles in AutoDesk MeshMixer to ensure proper operation of the system. Two types of 3D models were segmented for registration, one containing only the frontal face and head without the eyes, and one containing the full head without the eyes. The second model was used as a back-up registration target for registration. It contained a larger surface to register onto, but it also introduced more noise because the skin of the head was often covered by hair and therefore not fully visible for automatic depth-based registration. The model shown in Fig. 3b was only used and tried in case the facial surface of a patient was obstructed due to the placement of the Mayfield clamp, or due to needed positioning for optimal approach of the biopsy trajectory, leading to failed registration when using the model in Fig. 3a as reference. These models were segmented from the original-sized model skin surface model in AutoDesk MeshMixer as exemplified in Fig. 3. This segmented model was then used as a reference for surface-based registration.

#### Intraoperative observational experiment

The neurosurgical biopsies were performed with the use of the current clinical gold standard for frameless procedures, the Medtronic StealthStation S8 (cf. Table 1). This is an image-guided surgical navigation tool that uses infrared (IR) reflective spherical markers on a pointer probe for registration of the preoperatively planned biopsy trajectory to the live surgical situation. Neurosurgeons who executed the procedure planned a biopsy trajectory on the Medtronic StealthStation S8 as per protocol, where the entry point of the biopsy trajectory was placed on the skin, and the target point was placed on the target biopsy location. These coordinates were then exported and inputted into the ARCUS system and visualized in the virtual overlay. A rough estimation of the accuracy of the overlay could directly be visually assessed by checking the distance between the transparent virtual skin and the actual patient’s skin, specifically on the nose and forehead. If a large deviation in position or rotation was seen, we concluded a failed registration.

Each study was conducted after fixating the patient’s skull in a Mayfield clamp and after the registration of the pointer to the StealthStation, but before the start of actual biopsy procedure. The neurosurgeons used the common surgical threshold of 2.0 mm for the minimum needed registration accuracy of the StealthStation S8. The registration accuracy within the StealthStation system as provided in the registration step of the system was noted for each procedure. After four cases, it was noticed that reregistration was sometimes required due to tracking loss of the HMD. Consequently, spatial anchoring was added for the remainder of the inclusions [27]. This did not alter the registration method.

**Fig. 4:**
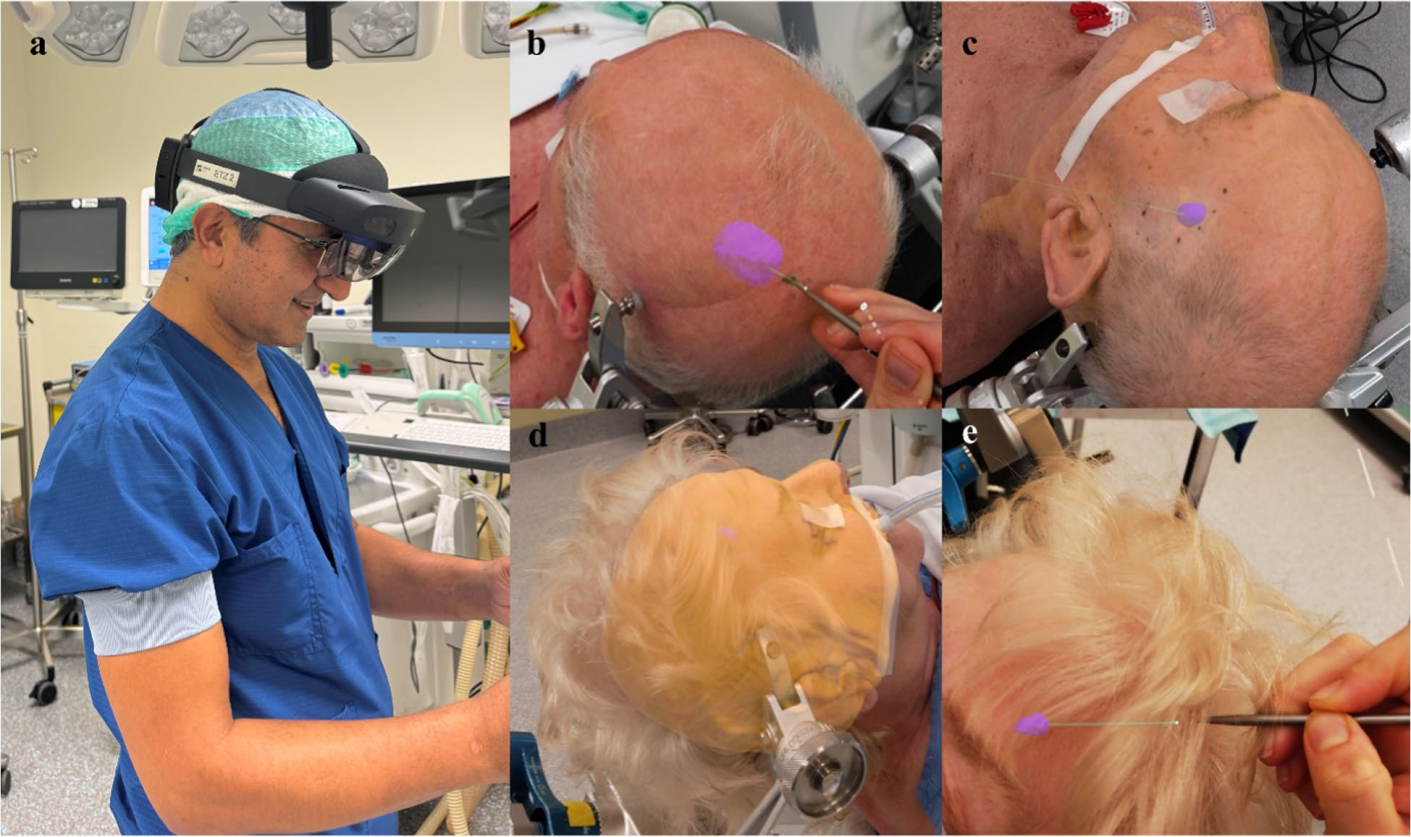
Examples of the experimental setup for the observational patient study. Once a patient had been secured into a Mayfield clamp, the surgeon used an HMD for the registration of the patient-specific 3D-models from the preoperative MR scan onto the face as shown in (**a**). Subfigures **b** and **c** show overlays on a male patient, where the skin is set to almost full transparency. The lesion (purple spot) and biopsy trajectory (green) are measured with a pointer. Subfigure **d** shows a holographic overlay onto a female patient with the skin set to almost fully opaque. Subfigure **e** shows the measurement of the biopsy trajectory accuracy by putting the pointer onto the holographic entry point (white) and aligning it with the trajectory (green). Note that (**b**-**e**) are the scenes viewed by the surgeon though the HMD device (**a**)

Once the registration with the StealthStation had been completed, the ARCUS system was used to create a precise overlay on the patient, visualizing the biopsy trajectory, and the 3D models segmented from the MR scan (Fig. 4). The skin surface hologram could be changed in opacity or toggled off entirely, depending on the preference of the user. For the actual measurements, the skin surface model was always disabled, so our system only showed the biopsy trajectory and, if applicable, the lesion at that moment. The planar passive blunt probe of the Medtronic StealthStation S8 was then held on the entry point position on the skin, which was displayed as a white sphere in the hologram. Subsequently, it was aligned with the virtually overlayed biopsy trajectory to achieve optimal orientation according to the AR-based guidance system. The “Off Plan” distance as displayed on the StealthStation was then used to measure the total offset from the holographic trajectory to the planned trajectory. This describes the shortest Euclidean distance between the probe tip and the planned trajectory. Since this research concerns solely observational measurements, only the “Off-plan” distance between the augmented reality-based overlayed entry point and the entry point calculated by the StealthStation was measured as the target point accuracy measurements would require invasive testing.

## Results

First of all, a phantom test was conducted to determine the actual registration accuracy and precision of the StealthStation between a planned point and the actual point at different given registration accuracies provided by the system itself. These are depicted in Table 2. The overall mean offset between the actual trajectory and the planned trajectory by the StealthStation was 1.2 mm. A Shapiro–Wilk test showed that this data was not normally distributed (*W* = 0.972, *p* < 0.001). Therefore, a Spearman’s rank correlation test was performed. Spearman’s ρ was found to be 0.304 (*p* < 0.01), indicating a weak to moderate, albeit significant correlation between the off-plan distance of the StealthStation and its initial within-system registration accuracy.

**Table 2:** The average measured off-plan distance per registration accuracy of the StealthStation navigation system

StealthStation Registration Accuracy (mm)	Detected Off-Plan Distance (mm)
0.5	0.9 ± 0.4
1.0	1.2 ± 0.5
1.5	1.1 ± 0.7
2.0	1.7 ± 0.6
2.5	1.2 ± 0.5

An observational experiment was performed on a total of 17 patients, of which the overlay did have spatial anchoring for 13 patients but 4 patients did not. This was because after 4 cases, tracking loss of the HMD was observed that resulted in the need for reregistration. Successful registration was achieved in 16 of 17 participants. One participant was excluded from analysis due to incorrect manual placement on the skull which resulted in an outlier value defined as 3SD above the mean. Thus, a total of 15 subjects were included for statistical analyses, still in line with sample sizes in similar studies (Table 1).

Eleven registrations were achieved with the face only as reference model, while four cases achieved registration with the full head as reference model. A Shapiro–Wilk test showed that the off-plan distance data was not normally distributed (*W* = 0.830, *p* < 0.01). A Wilcoxon rank resulted in a value of *W* = 11, *p* > 0.1, indicating no significant difference between the outcomes based on reference registration model. Moreover, out of the 15 included subjects, 6 were measured in supine neutral position and 9 were measured in supine rotated position. A Wilcoxon rank resulted in a value of W = 20, *p*-value = 0.4559, indicating no significant relation between patient position and measured offset.

**Fig. 5:**
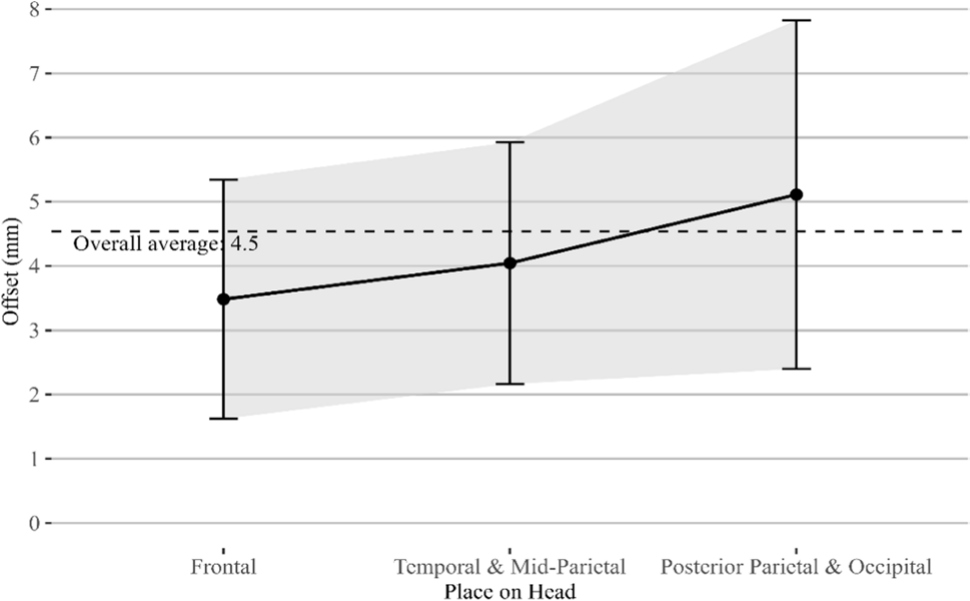
The Off-Plan distance of the ARCUS system as measured by the StealthStation for each area of the head. The standard deviation per location is denoted with error bars

The average “Off Plan” distance from the holographic trajectory to the trajectory planned by the StealthStation, as measured freehandedly with the passive navigation probe, was 4.5 ± 2.2 mm. The measured offset between the StealthStation biopsy trajectory and the holographic biopsy trajectory per location on the head is shown in Fig. 5 below. A Kruskal–Wallis test yielded no significant differences in offset between biopsy locations, *χ*^*2*^(2) = 1.117 with *p* > 0.5.

## Discussion

The current study used a markerless holographic surgical navigation system for neurosurgical biopsies, comparing the accuracy of the system with the clinical gold standard for frameless procedures. The first experiment compared the actual registration accuracy and precision of the clinical gold standard with the registration accuracy given by the system’s own output. The second experiment measured the offset between the proposed ARCUS system overlayed biopsy trajectory location and the location according to the clinical gold standard. Fifteen patients were included for statistical analysis, a sample size comparable to similar studies (e.g., Table 1). Our results showed that the average total clinical error of the measurements of the proposed algorithm on actual patients was 4.5 mm ± 2.2 mm. This is lower than the multiple total error values reported in prior research on the accuracy of the StealthStation, while maintaining a comparable standard deviation. Our reported total clinical error, however, does not only include the ARCUS system’s target registration error, but a at least eight different sub-errors, including:

1. The accuracy of the entry point placement of the planned trajectory onto the skin. This average accuracy was 0.9 mm, with ± 0.7 mm;
2. the offset between the skin entry point and the probe entry point due to the compressibility of the skin;
3. the skin shift due to the variation in position as described in Furuse et al. [7];
4. the accuracy of the StealthStation registration of the preoperative model to the patient’s head during surgery. The average accuracy of the Medtronic StealthStation registration was 1.2 mm;
5. the accuracy of the instrument position detection by the StealthStation;
6. the visualization error of the hologram by the AR HMD;
7. the accuracy of the manual, freehand placement of the pointer, both in position and orientation;
8. the accuracy of the registration by the ARCUS system.

As expected, a larger deviation in offset of the proposed ARCUS algorithm is seen when the biopsy location is further to the dorsal part of the brain. This observation is also described in Furuse et al., where a larger offset up to 9 mm is seen for use of gold standard navigation systems such as the StealthStation for lesions at the posterior area of the head, likely due to the skin shift phenomenon [7]. Moreover, a small possible angular error would translate to a larger Euclidean offset when a target is further from the registration surface. Above all, the ARCUS system achieves registration within 10 seconds, resulting in a significantly faster intraoperative setup compared to the 15-minute setup of current systems such as the StealthStation. However, preoperative steps were not compared since our proposed system is still in a research stage and automated preoperative segmentation was for now regarded out of scope for this study.

While our reported total clinical error may not yet reflect the desired sub-millimeter accuracy, we show improved performance to what has currently been reported for markerless systems. For instance, an earlier study on the proposed system reported accuracy of 12.4 ± 1.2 mm [9]. Gsaxner et al. reported an accuracy of 9.2 ± 1.5 mm for a system applicable to facial procedures in a phantom setting and one human setting [12]. Recent phantom-based research on markerless AR navigation by Kerkhof et al. shows mean offsets of 7.18 ± 4.19 mm [13].

The total clinical accuracy found in this research (4.5 ± 2.2 mm) is higher than the accuracies reported for the current clinical gold standard as shown in Table 1. Even though our standard deviation, also regarded as precision, may be larger than the reported results in Table 1, Girgis et al. report a standard deviation of 2.2 mm as well for the Medtronic StealthStation, which is comparable to our results [8]. Thus, the outcomes of this research demonstrate the potential of the proposed system based on entry point offset and encourage further research to support these results.

### Limitations

Despite the promising findings, any novel application is subject to limitations. Out of the 17 registration attempts, one attempt failed. This is likely due to the Mayfield clamp being attached right in the middle of the forehead for this particular patient, which distorted the depth data and prevented a proper global registration.

The results of the ARCUS system are in line with the findings in related studies, relating to the entry point offset in this observational study. Further interventional research is still needed to examine the precision and accuracy of the system within a full clinical setup, where not only the entry point offset but also the target point offset is considered. This is essential for further examining the operation of our system before it can be deemed suitable for clinical application.

Importantly, however, we would argue that the ARCUS findings reported in the current paper show an overestimation of the offset. As described earlier, the total measured clinical error includes various sub-errors that cannot be solely attributed to the ARCUS algorithm registration and visualization error. Due to the nature of the experiments conducted, it is hard to isolate the errors that were caused by the ARCUS system. For instance, the offset caused by the StealthStation system always included the offset of the ARCUS algorithm as well, skewing the data negatively. Nevertheless, this method was decided to be most beneficial for an observational patient study after extensive discussion with researchers and neurosurgeons. The results of our phantom study show that the registration accuracy generated by the StealthStation does not always reflect the actual accuracy. The conducted phantom tests and post-analyses of the point placement provide insight into these included offsets, yet do not allow for the exact estimation of the deviation added to the accuracy of the ARCUS algorithm reported here. We therefore estimate that the offset purely caused by the ARCUS system, is likely lower than the total offset reported in the current study, albeit a question for further investigation.

### Future perspectives

Future research in AR-based surgical navigation should focus on improving efficiency of the algorithm and on solving the above-mentioned limitations. First of all, to improve registration success rate, in future research we propose using not only the depth sensor but also the RGB camera. This camera would allow for both filtering out metal instruments and sterile covers, but also to integrate a basic facial recognition algorithm to help optimize the global registration process. Next, ease-of-use is expected to increase due to the fully automated registration method of the proposed system that achieves a registration within 6–10 seconds with no manual input. Setup time currently is under one minute, depending on how fast the operator puts the HMD on their head.

Future research may also include the use of stick-on measurement markings on a patient before their MRI scan, where those markings could be used to measure the offset of our overlayed hologram with respect to the marking while minimizing the introduction of additional errors. Moreover, other parameters such as stability of the hologram over time and after draping should be considered in future studies.

Finally, to improve efficiency of the ARCUS system, automated AI-based segmentation algorithms could be implemented for swift skin and lesion segmentation from the MRI scan. This would also make the system suitable for (semi)acute indications and for situations which need a segmentation of soft-tissue with varying proton characteristics which are not easily segmented by simple computer vision algorithms. Moreover, AI could be implemented for initial detection of relevant anatomical areas such as the face for faster convergence of the registration. The current findings provide a proof-of-concept for clinical use of an AR-based system for neuronavigation demonstrating promise for a safer and more efficient process in neurosurgical biopsies.

## Conclusion

This paper proposes ARCUS, a depth-based, markerless AR-based system for neurosurgical navigation. Initial research on patients shows an accuracy of the total clinical error comparable to the clinical gold standard for frameless procedures. Further research is still needed to prove full operability and reliability of the proposed system. Still, this initial observational in-patient research paves the way for a new navigation system that improves efficiency, ease-of-use and registration speed, offering high potential for neurosurgical biopsies.

## Data Availability

Data from this research will be shared with researchers upon methodologically sound and reasonable requests to achieve aims described in their approved requests, beginning 3 months and ending 5 years following article publication. Available data includes measurements results reported in this article, after de-identification, the study protocol, statistical analysis plan and analytic code. Raw data shall not be shared to protect patient privacy. Proposals should be directed to a.groenenberg@etz.nl; to gain access, data requestors will need to sign a data access agreement.
